# Gonorrhoea Diagnostic and Treatment Uncertainties: Risk Factors for Culture Negative Confirmation after Positive Nucleic Acid Amplification Tests

**DOI:** 10.1371/journal.pone.0155017

**Published:** 2016-05-06

**Authors:** Rebecka Vyth, Amy Leval, Björn Eriksson, Eva-Lena Ericson, Lena Marions, Maria-Pia Hergens

**Affiliations:** 1 Department of Communicable Disease Control and Prevention, Stockholm County, Stockholm, Sweden; 2 Department of Medicine, Infectious Disease Unit, Karolinska Institutet, Stockholm, Sweden; 3 Division of Clinical Microbiology, Karolinska University Hospital, Huddinge, Stockholm, Sweden; 4 Department of Clinical Science and Education, Karolinska Institutet, Södersjukhuset, Stockholm, Sweden; Midwestern University, UNITED STATES

## Abstract

Gonorrhoea incidence has increased substantially in Stockholm during the past years. These increases have coincided with changes in testing practice from solely culture-based to nucleic acid amplification tests (NAAT). Gonorrhoea NAAT is integrated with *Chlamydia trachomatis* testing and due to opportunistic screening for chlamydia, testing prevalence for gonorrhoea has increased substantially in the Stockholm population. The aim of this study was to examine epidemiological risk-factors for discordant case which are NAAT positive but culture negative. These discordant cases are especially problematic as they give rise to diagnostic and treatment uncertainties with risk for subsequent sequelae. All gonorrhoea cases from Stockholm county during 2011–2012 with at least one positive *N*. *gonorrhoea* NAAT test and follow-up cultures were included (N = 874). Data were analysed using multivariate and stratified logistic regression models. Results showed that women were 4-times more likely (OR 4.9; 95% CI 2.4–6.7) than men to have discordant cultures. Individuals tested for gonorrhoea without symptoms were 2.3 times more likely (95% CI 1.5–3.5) than those with symptoms to be discordant. NAAT method and having one week or more between NAAT and culture testing were also indicative of an increased likelihood for discordance. Using NAAT should be based on proper clinical or epidemiological indications and, when positive, followed-up with a culture-based test within one week if possible. Routine gonorrhoea testing is not recommended in low prevalence populations.

## Introduction

**Fig 1:**
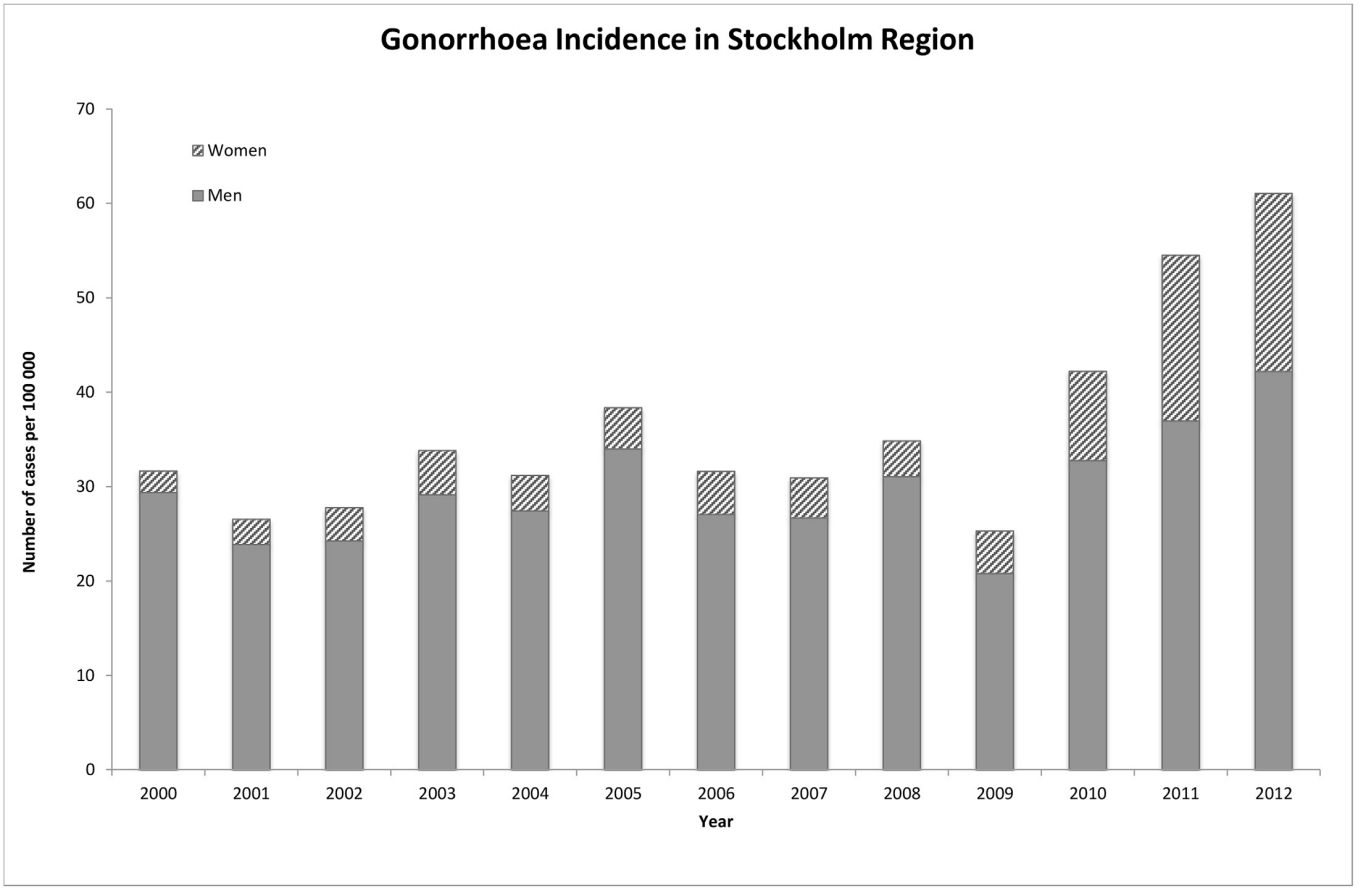
Trends in gonorrhoea, Stockholm region 2000–201.

Gonorrhoea incidence has increased substantially in Stockholm during the past years [1] “Fig 1”. The advent of Nucleic Acid Amplification Tests (NAAT) for *Neisseria gonorrhoeae* has led to a higher prevalence of gonorrhoea testing in the population with well over 100 000 tests annually, 70% of those among women. Due to ease of use and high test performance, NAATs could feasibly increase diagnostic yield, thereby helping control the spread of gonorrhoea. Before the introduction of NAAT in 2010, approximately 25 000 tests (cultures) were performed annually in the Stockholm region. NAATs are taken at a wide variety of primary care and outpatient clinics throughout the region. When positive, culture specimens are requested from specific clinics for sexually transmitted infections (STI) as Stockholm has a system of centralized referrals for gonorrhoea confirmation, treatment and contract tracing.

Among heterosexual women in Sweden, the number of reported cases increased by approximately 150% after the introduction of NAAT [2]. Increases in both heterosexual men and men having sex with men (MSM) have been 35%. In Sweden, the Communicable Diseases Act from 2004 states that gonorrhoea diagnosis entails mandatory reporting to communicable disease authorities with subsequent contact tracing required. Contract tracing requires individuals to name all sexual contacts potentially involved, thus the personal and social consequences of a diagnosis could be significant. Before treatment is initiated, it is recommended that antibiotic sensitivity of the isolate be determined using culture-based methods [3]. This is done in order to avoid ineffective treatments with inherent risks for patient sequelae and on a broader scale to prevent development and spread of multi-resistant bacteria.

However, a substantial portion of cases positive with NAAT cannot be verified by culture and hence it is not possible to determine resistance patterns. If these cases are treated, they often receive a standard treatment that may not be effective and potentially lead to an increase in antibiotic resistance [4]. In some countries, see and treat strategies are used due to fears that patients will not return for treatment if test positive [5]. If resistance patterns could be identified using molecular techniques the problem with inappropriate treatment would be more easily solved [6,7].

Uncertainty in diagnostics and treatment of NAAT positive, culture negative gonorrhoea can lead not only to physical complications but also to psycho-social complications and distress for those diagnosed. In this emerging field of NAAT-based testing for gonorrhoea, more knowledge is needed on epidemiological risk factors for testing result discordance so as to best inform public health practices optimal for individual infection care. Therefore, the objective of this study is to examine various epidemiological risk factors for NAAT positive but culture negative gonorrhoea cases to better understand these increasing incidence rate trends and result discordance.

## Materials and Methods

### Cohort

Data were obtained from Sweden’s national electronic surveillance system for the reporting of communicable diseases, SmiNet, where all gonorrhoea cases and suspected cases are reported. According to the National Board of Health and Welfare in 2008, cases of gonorrhoea were defined by the following criteria; 1) *N*. *gonorrhoeae* culture positive from a clinical sample or 2) *N*. *gonorrhoeae* NAAT positive in clinical a sample or 3) findings of Gram-negative intracellular diplococci from urethra smear from men [8]. As of 2012, *N*. *gonorrhoeae* NAAT positive in clinical sample had to be confirmed with a positive culture according to the guidelines from the National Board of Health and Welfare [9]. However, this confirmation requirement was not consistently followed. Every NAAT positive test was still reported to SmiNet regardless of the culture result. During the study period, January 1, 2011-December 31, 2012, 1236 cases of gonorrhoea were reported in Stockholm County. Cases were excluded (n = 362) if a) no NAAT (n = 332), b) no culture n = (9), c) antibiotic treatment after NAAT but before culture(n = 7) or d) NAAT from rectum, throat or eye, as the method was not approved by ISO-certification (n = 14). In total, 874 cases having at least one positive *N*. *gonorrhoeae* NAAT test and follow-up cultures were included. Data on sex, age, mode of transmission, NAAT-test location, symptoms, Chlamydia trachomatis co-infection two months prior to or after gonorrhoea diagnosis, reason for testing, culture location, days between NAAT and culture, and NAAT method were collected from clinical and laboratory data. Three different NAAT methods were in use in the county of Stockholm. The majority of samples were analysed by the Becton Dickinson technique using SDA, which is an isothermal technology utilized to generate billions of copies of target molecules from a single DNA template. The other methods are Roche Cobas 4800 utilizing amplification of target DNA by the Polymerase Chain Reaction (PCR) followed by nucleic acid hybridization, and the Abbott RealTime which is a polymerase chain reaction (PCR) assay for the direct, qualitative detection of the genomic DNA of *N*. *gonorrhoeae*. All three Stockholm laboratories report positive tests (NAATs and/or cultures) in SmiNet. Positive NAAT were not confirmed with second NAAT primers during the study period.

Clinicians are required to report data on mode of transmission (hetero or homosexual), presence of symptoms (yes/no) and reason for gonorrhoea testing (contact tracing, belonging to a high risk group or symptoms) into SmiNet. The definition of which individuals are considered as part of a ‘high risk group’ is not predefined and is up to reporting clinicians to define. Gonorrhoea cases are defined by reported clinical and laboratory data.

### Analyses

Descriptive statistics, including annual incidence trends per 100 000 were performed, stratified by sex. Main outcome variable is defined as a positive NAAT but negative follow-up culture (+-) and will hereafter be referred to as discordant cases. Univariate and multivariate logistic regression were performed where exposure covariates most likely to affect outcome were included in the univariate and multivariate models. Odds-ratios with ninety-five percent confidence intervals were assessed in the model building process. Multicollinearity was evaluated prior to constructing final models. As the variables “symptoms” and “reason for gonorrhoea testing” were collinear, only one of these variables was used in each of the final models. Three separate models were used for combined and sex stratified analyses: 1) univariate, 2) multivariate using the variable “symptoms” and excluding “reason for testing” and 3) multivariate using the variable “reason for testing” and excluding the variable “symptoms”. SAS Enterprise Guide version 5.1 was used for data management and analysis.

This study was granted ethical approval by the Regional Ethics Committee in Stockholm (Dnr 2013/1728-31/1-2013) for the use of routinely collected surveillance data. Surveillance routines for this specific disease area dictate that all records be collected without the use of a unique identifier. Prior to the analysis, steps were taken to further remove all possible personal identifiers and only anonymized and de-identified data were used in the analysis. No informed consent for these de-identified data was required by the ethical approval committee.

## Results

In total, 874 individuals were included in the study, 501 men and 373 women. Thirty-three percent of NAAT positive cases tested negative in the culture based method (n = 289) (Table 1). Fifty-one percent of the women with positive NAAT were discordant cases, while the corresponding number for men was 20%. Mean age was 24 for women and 31 for men. Individuals in the oldest age group had a lower proportion of discordant cases compared to the other age groups. No differences in discordant cases were seen between individuals with and without chlamydia co-infection. Among those who were symptomatic at testing, 22% were discordant cases. For those without symptoms the corresponding number was 51%. Individuals whose reason for testing was due to contract tracing had a higher proportion of discordant results (44%) compared to those tested due to symptoms (23%) or because they belonged to a risk group Abbot Real Time and Roche Cobas 4800 had results with lower discordance (24% and 23% respectively) compared to Becton Dickinson SDA (36%). Mean days between NAAT and culture were 7.5 for discordant cases and 3.5 for culture-positive. No differences in discordant cases were seen among heterosexual vs. homosexual transmission (17% respectively).

**Table 1:** Descriptive information on gonorrhea NAAT positive cases included in the study. 2011–2012.

	Culture neg n (%)^a^	Culture pos n (%)^a^	Total
**Total**	289	585	874
**Sex**			
Men	98 (20%)	403 (80%)	501
Women	191 (51%)	182 (49%)	373
**Age group**			
< 20 yrs	54 (39%)	86(61%)	140
20–29 yrs	151(35%)	287 (65%)	429
30–39 yrs	53 (32%)	113 (68%)	166
40- yrs	31 (22%)	108 (78%)	139
**Chlamydia co-infection**			
Yes	41 (30%)	95 (70%)	136
No	248 (34%)	490 (66%)	738
**Symptoms**			
Yes	131 (22%)	456 (78%)	587
No	123 (51%)	120 (49%)	243
Missing	35 (80%)	9 (20%)	44
**Reason for testing**			
Symptoms	117 (23%)	381 (77%)	498
Contact tracing	68 (44%)	86 (56%)	154
Risk group	33 (24%)	102 (76%)	135
**NAAT method**			
Becton Dickinson SDA	248 (36%)	449 (64%)	697
Abbott Real Time	16 (24%)	51 (76%)	67
Roche Cobas 4800	25 (23%)	83 (77%)	108
Missing	0	2 (100%)	2
**Days between NAAT and culture**			
< 7 days	108 (20%)	444 (80%)	552
7- days	103 (42%)	141 (58%)	244
Missing	78 (100%)		78
**Mode of transmission (among men)**^b^			
Homosexual transmission	36 (17%)	182 (83%)	218
Heterosexual transmission	45 (17%)	214 (83%)	259
Missing/other	17 (71%)	7 (29%)	24

^a^ Row percent shown

^b^ Among women there was no homosexual transmission reported

Women were four-times more likely than men to be discordant (OR 4.0; 95% CI 2.4–6.7) (Table 2, model 1). Individuals tested for gonorrhoea without symptoms were 2.3 times more likely than those with symptoms to be discordant (95% CI 1.5–3.5). NAAT methods Abbot Real Time (OR 0.4: 95% CI .2–0.9) and Roche Cobas 4800 (OR 0.3; 95% CI 0.1–0.5) were associated with a decreased risk of discordancy compared to Becton Dickinson SDA. One week or more between NAAT and culture testing was also indicative of an increased likelihood for discordant results. When adjusting for the variable “reason for testing” instead of the variable “symptoms”, differences between men and women increased substantially, indicating effect modification by sex and warranting a stratified model.

**Table 2:** Multivariate logistic regression modeling risks for gonorrhea NAAT positive, culture negative cases, presented as odds ratios with 95% confidence intervals.

		OR^a^	(95% CI)	OR^b^	(95% CI)	OR^c^	(95% CI)
**Sex**							
Men	98	ref		ref			
Women	191	4.3	(3.2–5.8)	4.0	(2.4–6.7)	5.3	(3.1–9.2)
**Age group**							
< 20 yrs	54	ref		ref			
20–29 yrs	151	0.9	(0.6–1.3)	1.6	(1.0–2.7)	1.8	(1.0–3.3)
30–39 yrs	53	0.7	(0.5–1.2)	1.8	(0.9–3.6)	1.8	(0.8–3.7)
40- yrs	31	0.5	(0.3–0.8)	1.6	(0.8–3.4)	1.6	(0.7–3.4)
**Chlamydia co-infection**							
No	248	ref		ref			
Yes	41	0.9	(0.6–1.3)	0.8	(0.5–1.4)	0.8	(0.5–1.4)
**Symptoms**							
Yes	131	ref		ref			
No	123	3.6	(2.6–4.9)	2.3	(1.5–3.5)		
**Reason for testing**							
Symptoms	117	Ref					
Contact tracing	68	2.6				2.5	(1.6–2.7)
Risk group	35	1.1				0.8	(0.4–1.3)
**NAAT method**							
Becton Dickinson SDA	248	ref		ref			
Abbott Real Time	16	0.6	(0.3–1.0)	0.4	(0.2–0.9)	0.4	(0.2–1.0)
Roche Cobas 4800	25	0.5	(0.3–0.9)	0.3	(0.1–0.5)	0.3	(0.1–0.6)
**Days between NAAT and culture**							
< 7 days	108	ref		ref			
7- days	103	3.0	(2.2–4.2)	2.6	(1.7–4.0)	2.5	(1.6–3.9)
**Mode of transmission**							
Heterosexual transmission	45	ref		ref			
Homosexual transmission	36	0.4	(0.2–0.5)	1.1	(0.6–2.7)	1.6	(0.8–2.9)

^a^ Univariate model

^b^ Multivariate model including, sex, age group, chlamydia co-infection, symptoms, NAAT method, days between NAAT and culture and mode of transmission

^c^ Multivariate model including, sex, age group, chlamydia co-infection, reason for testing, NAAT method, days between NAAT and culture and mode of transmission

When stratifying analyses based on sex, men without symptoms had significantly increased risk for discordant results (OR 6.6; 95% CI 3.3–13.3) while symptoms were not predictive for women (OR 1.4; 95% CI 0.8–2.2) (Table 3). In contrast, there was decreased risk for women being discordant when contact tracing (as opposed to symptoms) was the reason for testing (OR 0.4; 95% CI 0.2–0.8). This effect was not seen among men (OR 2.1 95% CI 0.9–5.1). One week or more between NAAT and culture testing were indicative of an increased likelihood for discordant results among men (OR 6.3; 95% CI 3.0–13.1) but less so among women (OR 1.7; 95% CI 1.0–2.8).

**Table 3:** Stratified multivariate analyses modeling risks for gonorrhea NAAT positive, culture negative cases, presented as odds ratios with 95% confidence intervals.

	WOMEN				MEN			
	Nr^a^	OR^a^ (95%CI)	OR^c^(95%CI)	OR^d^(95%CI)	Nr^a^	OR^b^ (95%CI)	OR^c^(95%CI)	OR^d^(95%CI)
**Age group**								
< 20 yrs	49	ref	ref	ref	5	ref	ref	ref
20–29 yrs	109	1.5 (1.0–2.4)	1.6 (0.9–2.8)	2.3 (1.1–3.7)	42	1.1 (0.4–3.1)	1.8 (0.3–9.2)	1.7 (0.3–8.6)
30–39 yrs	23	1.8 (0.9–3.8)	1.7 (0.7–4.0)	1.5 (1.5–4.3)	30	1.5 (0.5–4.2)	2.4 (0.4–13.4)	2.0 (0.4–10.9)
40- yrs	10	0.9 (0.4–2.2)	0.6 (0.2–1.9)	0.8 (0.2–2.8)	21	1.1 (0.4–3.1)	3.1 (0.6–17.4)	2.5 (0.5–13.3)
**Chlamydia co-infection**								
No	165	ref	ref	ref	83	ref	ref	ref
Yes	26	0.6 (0.4–1.1)	0.7 (0.3–1.3)	0.8 (0.4–1.65)	15	1.1 (0.6–2.0)	1.0 (0.4–2.3)	0.8 (0.3–1.9)
**Symptoms**								
Yes	82	ref	ref		49	ref	ref	
No	87	1.4 (0.9–2.1)	1.4 (0.8–2.2)		36	6.1 (3.6–10.4)	6.6 (3.3–13.3)	
**Reason for testing**								
Symptoms	72	ref		ref	45	ref		ref
Contact tracing	20	0.4 (0.2–0.7)		0.4 (0.2–0.8)	13	1.9 (1.0–3.9)		2.1 (0.9–5.1)
Risk group	48	1.2 (0.7–2.0)		1.2 (0.6–2.2)	20	3.1 (1.7–5.7)		3.0 (1.3–6.6)
**NAAT method**								
Becton Dickinson SDA	161	ref	ref	ref	87	ref	ref	ref
Roche Cobas 4800	17	0.3 (0.2–0.5)	0.2(0.1–0.4)	0.2(0.1–0.5)	8	0.3 (0.1–1.1)	0.7 (0.2–2.3)	0.7 (0.2–2.2)
Abbott Real Time	13	0.5 (0.3–1.1)	0.5 (0.2–1.2)	0.5 (0.2–1.4)	3	0.8 (0.4–1.7)	0.3 (0.1–1.5)	0.2 (0.02–1.7)
**Days between NAAT and culture**								
< 7 days	63	ref	ref	ref	45	ref	ref	ref
7- days	78	1.3 (0.9–2.1)	1.7 (1.0–2.8)	1.5 (0.9–2.6)	25	3.7 (2.1–6.5)	6.3 (3.0–13.1)	5.9 (2.8–12.3)
Mode of transmission*								
Heterosexual transmission					45	ref	ref	ref
Homosexual transmission					36	0.9 (0.6–1.5)	1.2 (0.6–2.6)	1.9 (0.9–3.9)

^a^ Number of cases

^b^Univariate model

^c^ Multivariate model including, sex, age group, chlamydia co-infection, symptoms, NAAT method, days between NAAT and culture and mode of transmission

^d^ Multivariate model including, sex, age group, chlamydia co-infection, reason for testing, NAAT method, days between NAAT and culture and mode of transmission

* Among women there were no homosexual transmission reported

## Discussion

This is the first study that examines epidemiological risk factors among gonorrhoea cases for culture negative confirmation after positive NAAT. The increased testing with NAAT has given rise to uncertainty in gonorrhoea diagnostics and risk for incorrect treatment among culture negative cases. Women are four times more likely than men to have discordant results. Men with symptoms were more likely to have culture positive confirmation tests. However for women, symptoms were not related to positive confirmations. Women were most likely to have conclusive results when they were tested due to contact tracing, a finding not true for men. These pronounced sex and symptom based differences warrant a review of testing practices.

Biological factors may account for some of these sex differences. *N*. *gonorrhoeae* is one member of the genus Neisseria. Other Neisseria species (*N*. *lactamica* or *N*. *subflava* for example) can exist in vaginal normal flora and are not pathogenic but can cross-react with *N*. *gonorrhoeae*, thereby lowering NAAT specificity for women depending on circulating genus-types in the population tested [10,11]. The BD Probe Tech, used as one of three Stockholm laboratory methods shows increased risk for cross reaction with *N*. *lactamica* [11].Tests analysed with BD Probe Tech had an increased risk for culture-negative results in our study which could corroborate this cross-reaction risk. Our study excluded tests from non-genital locations as NAAT are location-sensitive and if taken from the oropharynx for example, positive results potentially reflect a cross-reaction [11].

Between 90–95% of men contracting gonorrhoea develop genital symptoms of which the most common is urethritis, dysuria and/or purulent discharge within a week after exposure [12,13]. Men discovering discharge are more likely to seek medical help, whereas women having daily discharge would be less observant to subtle changes. This is clearly substantiated by our study which shows asymptomatic men are six-times more likely to have discordant results. For women, genital symptoms such as dysuria and cervicitis are indicative of various STIs or lower urinary tract infections [12,13]. However, over 50% with gonococcal cervicitis are entirely asymptomatic [13]. This would explain why symptoms among women were not associated with culture-positive results in our study. As with other bacteria, could it be that individuals without symptoms but with positive NAAT are merely transient carriers of gonorrhoea DNA and therefore have negative culture results? Or could it be that some individuals actually clear the infection but may have enough gonococcal DNA for a positive NAAT test? Our study has brought these questions to our attention but it was not possible for us to address them with available data.

Neisseria are fastidious bacteria and the time from testing to laboratory processing is crucial. Viability drops rapidly within hours after sampling and prompt transportation and proper storage is recommended [14]. One study on transport revealed only 67% of the positive NAATs (BD Probe Tec) was confirmed by culture [14]. Transport time between Stockholm clinics and laboratories is minimal (<24 hours) due to well-established routines. We were not able to specifically measure transport time but we have no reason to believe that transport time varies between culture-negative and culture-positive cases.

Concerns have been voiced about a high proportion of false positives for gonorrhoea NAAT screening, especially in a low prevalence population [6,15,16]. The relationship between specificity, disease prevalence and positive predictive value, makes it important to take positive predictive value into account prior to wide-spread testing. The estimated prevalence for gonorrhoea in the general population is less than 1%, with the estimated prevalence rising to 15–35% among high risk groups such as MSM [10,17,18]. Men tested in our study were more likely part of a high risk group, compared to women.

In one report positive predictive value was calculated to be only 77% assuming a test specificity of 99.7% in a population with a prevalence of 1% [19]. CDC and Public Health England only recommend screening for a gonorrhoea infection if the positive predictive value is greater or equal to 90% [20]. One study suggests a high cut-off value for NAAT, as low level positive results need to be interpreted with caution because, in that study, none of the low level results were confirmed with culture or confirmatory NAAT [15]. In our study, there were considerably more women than men being tested during the study period (87 228 women and 41896 men). When first introduced, the gonorrhoea NAAT was co-analyzed with the existing chlamydia test. These tests were routinely offered those visiting youth clinics, the vast majority of visitors being women. When testing women without specified indications, the risk of low positive predictive value increases.

The use of a second NAAT with different DNA targets is recommended to verify a positive NAAT for gonorrhoea [21]. This is estimated to increase the positive predictive value to near 100% [19]. Dual NAAT or confirmatory testing is not yet standard practice in this emerging testing field, partly due to feasibility and costs [10,22]. However, this routine is now implemented by the microbiological laboratories in the Stockholm region. This, in addition to specified indications for screening in the population, would increase the positive predictive value.

The study has several limitations. There is no clear definition for the terms ‘risk group’ or ‘symptoms’ in SmiNet. Individual clinicians may define those terms differently between men and women. Sample locations also differ between men and women. Therefore, stratifying by sex will account for any misclassification of exposure or differences in culture sample location. Our study includes different population groups including women and men below 23 years of age attending a youth clinic, those attending sexual health clinics, men who have sex with men attending specialized out-patient clinics, and patients seeing their local GP. However, culture samples are exclusively taken at sexual health and MSM clinics, which limit discrepancies in test routines including transportation.

This study shows that women tested for reasons other than contact tracing, and men tested without symptoms, have substantially increased risks for test method discrepancies. This puts these individuals at jeopardy for indeterminate diagnosis and/or improper treatment of gonorrhoea as well as unnecessary contact tracing. The use of NAATs should be based on proper clinical and epidemiological indications, such as symptoms, contact tracing, sex in countries with higher incidence of gonorrhoea and men having sex with men. Positive NAAT should be followed by a culture-based test, preferably within one week. Confirmation tests, using second NAAT primers is recommended, especially in low prevalence population groups.
